# Integrated transcriptomic and phenotypic analyses reveal spatiotemporal variation in neutrophil *Cd14* expression after hepatectomy

**DOI:** 10.1371/journal.pone.0356246

**Published:** 2026-08-18

**Authors:** Ahmad Adawy, Masaki Honda, Daiki Yoshii, Hiroki Hirao, Masahiro Tomita, Keita Shimata, Yoshihiro Komohara, Taizo Hibi

**Affiliations:** 1 Department of Pediatric Surgery and Transplantation, Kumamoto University Graduate School of Medical Sciences, Kumamoto, Japan; 2 Department of Cell Pathology, Graduate School of Medical Sciences, Kumamoto University, Kumamoto, Japan; 3 Department of Pediatric Surgery, Faculty of Medicine, Mansoura University, Egypt; Kaohsiung Medical University Hospital, TAIWAN

## Abstract

Neutrophils are key mediators of both hepatic injury and repair. This study aimed to characterize the spatiotemporal dynamics of Cd14 expression in neutrophils following hepatectomy and to explore its potential role in liver regeneration. We performed an integrated transcriptomic and phenotypic analysis of neutrophil Cd14 expression using bioinformatics and flow cytometry in immune cells isolated from murine livers after 70% partial hepatectomy. Cd14 ranked among the top 10 markers distinguishing activated liver neutrophils from quiescent or stimulated non-hepatic neutrophils. Both Cd14 mRNA and CD14 protein levels were significantly upregulated in hepatic neutrophils, peaking at 24 h after hepatectomy (p = 0.012). Gene set enrichment analysis revealed that Cd14 was associated with Toll-like receptor 4 and IL-6/JAK/STAT3 signaling pathways—both implicated in hepatocyte proliferation—suggesting that Cd14 serves as a marker of a pro-regenerative neutrophil phenotype and represents a potential therapeutic target to enhance liver regeneration following hepatectomy.

## Introduction

Unlike other epithelial tissues that depend on resident stem cells for regeneration, the liver relies on the proliferative capacities of distinct hepatocyte populations that vary in their regenerative potential under physiological and pathological conditions [1]. Hepatocytes possess remarkable and lifelong self-renewal capacity, enabling the liver to maintain a relatively “young” cellular age—estimated at less than 3 years—even in elderly individuals [2]. This regenerative ability underpins the success of extensive liver resections as well as partial and split liver transplantation. Furthermore, emerging evidence suggests that defective liver regeneration (DLR), rather than ischemia/reperfusion (I/R) injury, may play a crucial role in the development of small-for-size syndrome following hepatectomy or transplantation [3].

Both innate and adaptive immune cells, including neutrophils, contribute to the priming and proliferative phases of liver regeneration (LR) [1]. Beyond their classic role in pathogen clearance and NETosis, neutrophils are transcriptionally active and exhibit functional plasticity, adopting distinct phenotypes such as proinflammatory, anti-inflammatory, tumor suppressive, or pro-regenerative [4]. However, their precise role in LR remains incompletely defined. Evidence from neutropenic mouse models indicates that neutrophils facilitate the priming phase of LR through pathogen clearance and mitigation of oxidative stress, while supporting the proliferative phase via angiogenesis and secretion of hepatic growth factors [5]. In contrast, excessive intrahepatic neutrophil infiltration and excessive extracellular trap formation post-hepatectomy (PHx) can trigger overwhelming inflammation, contributing to DLR and acute liver failure [6,7]. Temporal heterogeneity in neutrophil phenotypes has been observed PHx [8], and recent studies have highlighted their dichotomous, time-dependent roles in the regeneration process [9].

In this study, we investigated the spatiotemporal dynamics of cluster of differentiation 14 (*Cd14*) expression in neutrophils PHx, as it is among the top 10 markers of activated hepatic neutrophils. Furthermore, we explored its potential role in LR through TLR4 and IL-6/JAK/STAT3 signaling pathways, which are known to enhance LR [10,11].

## Materials and methods

### Bioinformatics analysis

#### Gene expression omnibus online datasets [free access].

(1) Accession number: GSE137539

Single cell RNA sequencing (scRNA-seq) data were obtained from Boston children’s hospital, MA, USA. This dataset includes immune cells isolated from peripheral blood, bone marrow, liver, spleen and peritoneum under steady-state conditions and 24 h after Escherichia coli (E. coli) challenge.

(2) Accession number: GSE151309

scRNA-seq data were obtained from University of Illinois, IL, USA. This dataset comprises single cells isolated from mice livers at 24, 48, and 72 h following a two-thirds partial hepatectomy, as well as from untreated control mice.

(3) Accession number: GSE231391

Bulk RNA-seq data were obtained from Mayo clinic, MN, USA. This dataset includes bulk liver samples isolated from mice livers at 40 h following 70% partial hepatectomy, as well as from untreated control mice.

(4) Accession number: GSE278987

Bulk RNA-seq data were obtained from Sheba medical center, Israel. This dataset includes granulocyte myeloid-derived suppressor cells (G-MDSCs) isolated from mice livers at 1, 2, 3, and 4 days following 70% partial hepatectomy, as well as from sham-operated mice.

(5) Accession number: GSE211370

Bulk RNA-seq data were obtained from The Rockefeller University, NY, USA. This dataset includes bulk liver samples isolated from mice livers at 72 h following 70% partial hepatectomy, as well as from untreated control mice.

### scRNA-seq analysis

R 4.4.2 (2024-10-31 ucrt) and RStudio (2025.5.0.496) software were used for bioinformatics and other statistical analyses. The ‘Seurat’ package (version 5.3.0) was used for scRNA-seq analysis. Data obtained from Boston children’s hospital were already filtered and clustered; this dataset was subsetted to focus specifically on neutrophils, excluding other immune cell populations. Feature plots and violin plots were then used to investigate the *Cd14* expression across various neutrophil clusters.

In contrast, regarding the data from the University of Illinois, initial quality control was performed to exclude potential doublets (cells with > 4000 detected gene) and low-quality cells (with <200 detected genes or >5% mitochondrial gene content). Following normalization, identification of highly variable features, and data scaling, principal component analysis was run for linear dimensionality reduction. 50 principal components and a resolution parameter of 2.0 were used for clustering and dimensionality reduction via uniform manifold approximation and projection (UMAP). The ‘FindMarkers’ function was then applied to identify marker genes for each cluster (S1 Fig. A).

In addition to feature and violin plots, the ‘muscat’ package (version 1.18.0) was used for pseudo-bulk count aggregation. The aggregated counts were subsequently visualized using a heatmap and Venn diagram, generated with the ‘Pheatmap’ package (version 1.0.13), and ‘VennDiagram’ package (version 1.7. 3), respectively.

### Bulk RNA-seq analysis

The ‘DESeq2’ package version (1.44.0) was used for bulk RNA-seq analysis. Briefly, the ‘DESeq’ function was applied to perform differential gene expression analysis. Genes with very low expression (base mean <20) were excluded. Differentially expressed genes were defined using log_2_ fold change (log_2_FC > 1), and adjusted *P*-value (*P*adj < 0.05) for downstream visualization including volcano plots. ‘org.Hs.e.g.,db’ package version (3.19.1) and ‘AnnotationDbi’ package version (1.66.0) were used for gene annotation.

### Gene set enrichment analysis (GSEA)

GSEA was performed using the web-based tool developed by the Ma’ayan Lab of the Icahn School of Medicine at Mount Sinai, NY, USA (https://maayanlab.cloud/Enrichr/), as described before [12]. Various databases were explored for the *Ly6g–Cd14* gene pair, with the Gene Ontology Biological Process 2023 dataset (GO Biological Process 2023) and the Hallmark gene set collection 2020 from the Molecular Signatures database (MSigDB Hallmark 2020) selected for further analysis. The ‘enrichR’ R package (version 3.4) was used to retrieve enrichment results from these databases, and data visualization was conducted using the ‘ggplot2’ package (version 3.5.1).

### Animals

Wild-type C57BL/6 mice were purchased from Japan SLC, Inc. (Shizuoka, Japan) (http://www.jslc.co.jp/english/). Mice were bred in a specific pathogen-free environment under standardized conditions of temperature (21–22 °C) and a 12/12 h light/dark illumination cycle, with up to five mice per cage, at the Center for Animal Resources and Development (CARD, Kumamoto University, Japan), with ad libitum access to food and water. Only male, matched-weight mice aged 8–12 weeks were used for hepatectomy experiments, as liver regeneration is known to be impaired in female mice [13]. All animals received human care and all experiments were approved by Kumamoto University Ethics Review Committee for animal experiments (approval number: A2023-145).

### 70% partial hepatectomy

Isoflurane (Viatris Pharma, Tokyo, Japan) was used for inhalation anesthesia using a vaporizer (SN-487-0T; Shinano Manufacturing Co., Ltd., Tokyo, Japan) for all experiments. Mice were placed on a heating pad to maintain a body temperature of 37°C. After proper shaving and disinfection, laparotomy was performed through a 3 cm midline incision from the xiphoid process distally, and 70% partial hepatectomy was carried out using the clip technique as described previously [14], resecting the left lateral lobe and both left and right portions of the median lobe together with the gallbladder, leaving only the right lobe and caudate lobe intact. After intraperitoneal fluid resuscitation, the abdominal wall was closed in layers. For survival experiments, mice were allowed to recover from anesthesia and were monitored using the mouse body condition score for major liver resection [15]. For non-survival experiments, when blood, bone marrow, spleen or liver samples were collected for flow cytometry and/or immunohistochemical staining, euthanasia was performed using an overdose of isoflurane.

### Isolation of immune cells for flow cytometry

#### I. Liver samples.

A single-channel MINIPLUS 3 peristaltic pump (GLISON Inc., WI, USA) was used for retrograde perfusion of the liver through the vena cava, with periodical clamping in the portal vein, using type IV collagenase (Liberase). Corning® Hank’s Balanced Salt Solution (HBSS), 1X with calcium and magnesium (21–020-CV, Mediatech, Inc., VA, USA), Corning® HBSS, 1X without calcium, magnesium and phenol red (21–022-CV, Mediatech, Inc., VA, USA), HEPES solution (H0887-100ML, Sigma-Aldrich Corporation, MO, USA), and Liberase ™ Research Grade (05401127001, Roche Diagnostics GmbH, Mannheim, Germany) were used to prepare perfusion and digestion buffers, as previously described [16]. A TM-1A Thermax Water Bath (As One Co., Ltd., Osaka, Japan) was used to warm the digestion buffers to ensure optimal Liberase enzymatic activity for dissociate the extracellular matrix and retrieving a mixed suspension of liver cells. To obtain a high-quality single-cell suspension of leucocytes for flow cytometry, 40 um cell strainers, Percoll™ density gradient media (17089101, Cytiva Sweden AB, Uppsala, Sweden) and Gibco™ ACK Lysing Buffer (A10492-01, Life Technologies Corporation, NY, USA) were used for filtering cell debris and clumps, differential centrifugation and red blood cell lysis, respectively, as previously described [17].

#### II. Blood samples.

Blood samples were collected by cardiac puncture into heparinized tubes to obtain a sufficient volume for flow cytometry. Gibco™ ACK Lysing Buffer (REF: A10492-01, Life Technologies Corporation, NY, USA) was then used to lyse red blood cell and isolate a high-quality single-cell suspension of leucocytes for flow cytometry.

#### III. Bone marrow samples.

Upon euthanasia, the femur was dissected, and both the proximal and distal ends were transected. The bone marrow was then flushed from the shaft into a collecting tube using PBS. Gibco™ ACK Lysing Buffer (REF: A10492-01, Life Technologies Corporation, NY, USA) was subsequently used to lyse red blood cell and isolate a high-quality single-cell suspension of leucocytes for flow cytometry.

### Flow cytometry

After isolating a high-quality single-cell suspension of leucocytes, InVivoMAb anti‑mouse CD16/CD32 antibody (clone 2.4G2, catalog # BE0307), (Bio X Cell, NH, USA) was used first to block non-specific antibody binding for 15 minutes. Subsequently, cells were incubated with a fluorescent antibody cocktail for multicolor flow cytometry, while single-stained splenocyte controls were used for spectral compensation. To ensure data quality, a viability dye was applied to exclude dead or damaged cells. CD14 expression was double-validated using both isotype controls and fluorescence-minus-one controls. The gating strategy is illustrated in (S1 Fig. B).The following antibodies were used (Thermo Fisher Scientific, MA, USA):CD45 Monoclonal Ab (clone 30-F11), PerCP‑Cyanine5.5 conjugate (catalog #45‑0451‑82),CD45 Monoclonal Ab (clone 30-F11), APC conjugate (catalog #47‑0451‑82),CD45 Monoclonal Ab (clone 30-F11), FITC conjugate (catalog #11‑0451‑85),CD45 Monoclonal Ab (clone 30-F11), PE conjugate (catalog #12‑0451‑82), CD11b Monoclonal Ab (clone M1/70), APC conjugate (catalog #17-0112-82), CD14 Monoclonal Ab (clone Sa2−8), PE conjugate (catalog #12-0141-82), Rat IgG2a kappa Isotype Control (clone eBR2a), PE conjugate (catalog #12-4321-82),and, Fixable Viability Dye eFluor 780 (catalog #65-0865-14).Additionally, Ly-6G Monoclonal Ab (clone 1A8), FITC conjugate (catalog #127606), (BioLegend, Tokyo, Japan) was used as well.

Flow cytometry was performed using the SH800S Cell Sorter (Sony Biotechnology Inc., San Jose, CA, USA), and data were analyzed with FlowJo™ v10.10.0 (FlowJo, LLC, Ashland, Oregon, USA).

### Immunohistochemical staining

To investigate neutrophil density, liver samples obtained from mice after 70% partial hepatectomy were fixed in formalin and embedded in paraffin. Sections of 3-μm thickness were prepared using a microtome. After deparaffinization and heat-mediated antigen retrieval, endogenous peroxidase and non-specific binding were appropriately blocked. The slides were then incubated overnight at 4°C with anti-mouse monoclonal anti-Ly6g [EPR22909−135] (ab238132), purchased from Abcam plc (Tokyo, Japan), as the primary antibody. After washing, Histofine Simple Stain Mouse MAX-PO(R) secondary antibody for anti-rabbit primary antibody (code: 414341, Nichirei Biosciences, Tokyo, Japan) was used as the secondary antibody for 30 minutes at room temperature. A Histofine DAB (diaminobenzidine) substrate kit (code: 425011, Nichirei Biosciences, Tokyo, Japan) was used to visualize positive signals. Finally, the IHC-stained slides were examined by two expert pathologists to identify neutrophils.

### Statistical analysis

GraphPad Prism 9.5.1 (733) (GraphPad Software, LLC, San Diego, California), USA was used for was used to perform Student’s *t*-test and one-way analysis of variance (ANOVA). *P*-values < 0.05 were considered statistically significant.

## Results

### Upregulation of neutrophil *Cd14* transcript levels at 24–48 h PHx

**Fig 1:**
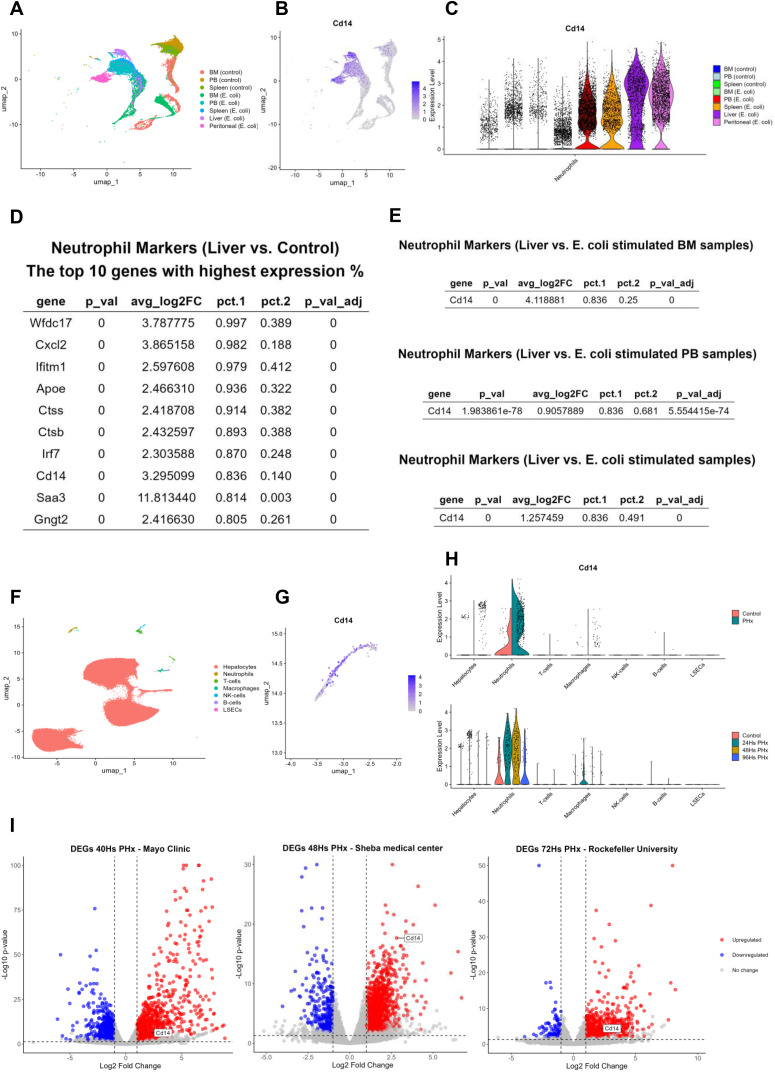
Transcriptomic analysis of neutrophil *Cd14* expression I. (A) Uniform Manifold Approximation and Projection (UMAP) of neutrophils annotated by tissue of origin and condition. (B) Feature plot and (C) violin plot of *Cd14* expression 24 h after E. coli challenge. (D) Top 10 markers of liver neutrophils 24 h after E. coli challenge. (E) Cd14 expression in liver versus non-hepatic neutrophils. (F) UMAP of single liver cells annotated by cell type. (G) Feature plot and (H) violin plot of *Cd14* expression 24, 48, and 72 h after hepatectomy. (I) Volcano plots of liver samples 40, 48, and 72 h after hepatectomy.

To characterize the transcriptional features of hepatic neutrophils, we first analyzed a comprehensive dataset from Boston Children’s Hospital that profiles neutrophil transcriptomic heterogeneity across multiple tissues under hemostatic conditions and 24h after E. coli challenge. This analysis revealed distinct genetic signature between liver and non-hepatic neutrophils (Fig 1A). *Cd14* was identified among the top 10 markers of activated hepatic neutrophils, exhibiting the highest transcript abundance. Notably 83.6% of hepatic neutrophils expressed *Cd14*, compared with only 14%, 25%, and 68.1% of quiescent, activated bone marrow, and peripheral blood neutrophils, respectively (Fig 1B–E). scRNA-seq data from the University of Illinois demonstrated a marked upregulation of *Cd14* expression in neutrophils at 24–48 h after two-thirds partial hepatectomy, with expression levels returning toward baseline by 96 h (Fig 1F–H). Consistently, bulk RNA-seq analyses of hepatic tissue samples collected at 40, 48, and 72 h PHx revealed upregulation of *Cd14* in pooled liver samples as well as in granulocytic myeloid-derived suppressor cells (Fig 1I).

**Fig 2:**
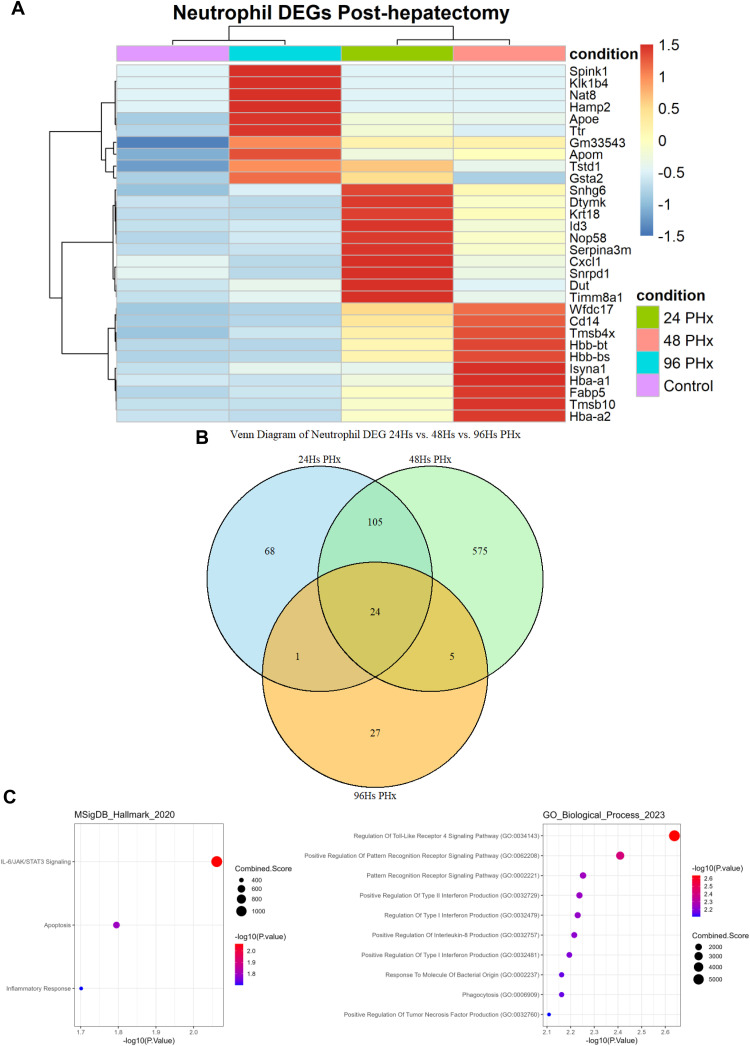
Transcriptomic analysis of neutrophil Cd14 expression II. (A) Heatmap of neutrophil top differentially expressed genes 24, 48, and 72 h after hepatectomy versus control. (B) Venn diagram of neutrophil differentially expressed genes 24, 48, and 72 h after hepatectomy. (C) Gene set enrichment analysis of the *Ly6g–Cd14* gene pair.

Hierarchical clustering of neutrophil transcriptomic profiles further supported these findings: neutrophils isolated at 24 and 48 h PHx clustered together, whereas those from control and 96 h samples formed a separate cluster, indicating similar gene expression patterns within each group (Fig 2A). Moreover, a Venn diagram of differentially expressed genes (DEGs) across the three time points showed that the 24- and 48-h groups exhibited the greatest number of DEGs, including numerous shared upregulated genes, while the 96-h group displayed relatively few DEGs and minimal overlap with the earlier time points (Fig 2B). To explore the functional significance of *Cd14* in neutrophils, GSEA was performed for *Ly6g–Cd14* gene pair. The analysis revealed enrichment of key signaling pathways associated with hepatocyte proliferation, including the Toll-like receptor 4 (TLR4) and IL-6/JAK/STAT3 pathways (Fig 2C), suggesting a potential role of *Cd14* in promoting LR PHx.

### Upregulation of neutrophil CD14 protein expression at 24 h PHx

**Fig 3:**
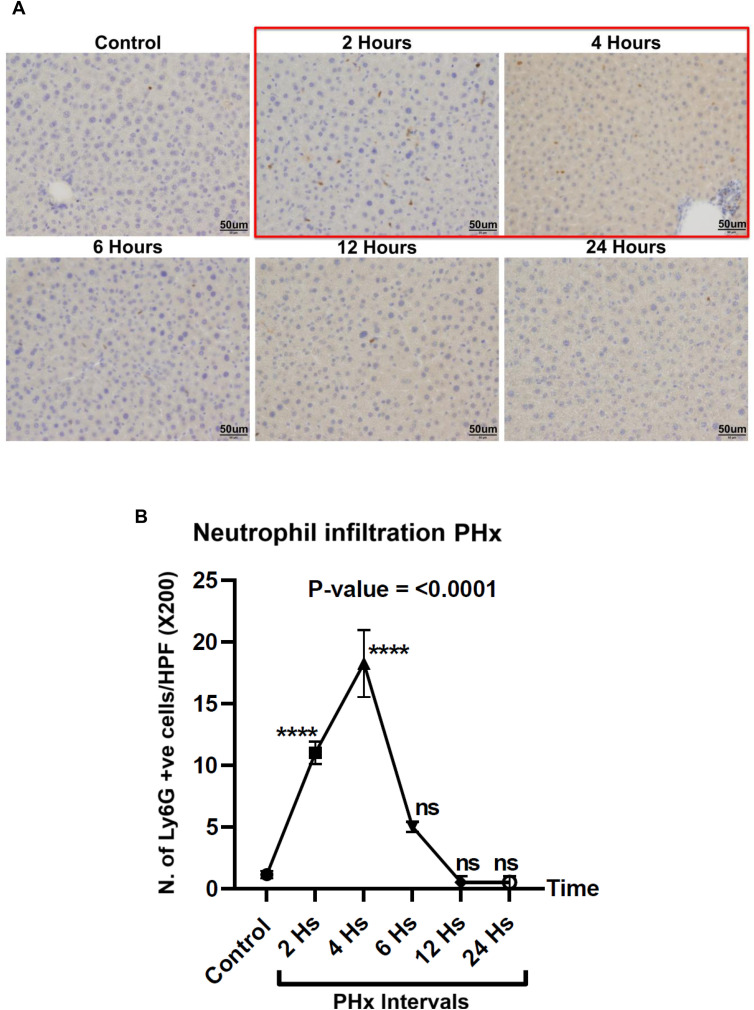
Quantitative analysis of neutrophil infiltration after hepatectomy I (A) Immunohistochemical staining of neutrophils 2, 4, 6, 12 and 24 h after hepatectomy. (B) Diagram summarizes temporal variations of neutrophil density 2, 4, 6, 12 and 24 h after hepatectomy (ANOVA).

To validate the transcriptional changes of *Cd14* at the protein level, 70% partial hepatectomy was performed in 8–10 week-old WT C57BL/6 male mice, followed by exploratory immunohistochemical (IHC) analysis. IHC revealed marked hepatic neutrophil infiltration as early as 2–4 h PHx (*p* < 0.001), whereas neutrophils were scarcely detectable at later time points (6–24 h PHx; Fig 3A–B).

**Fig 4:**
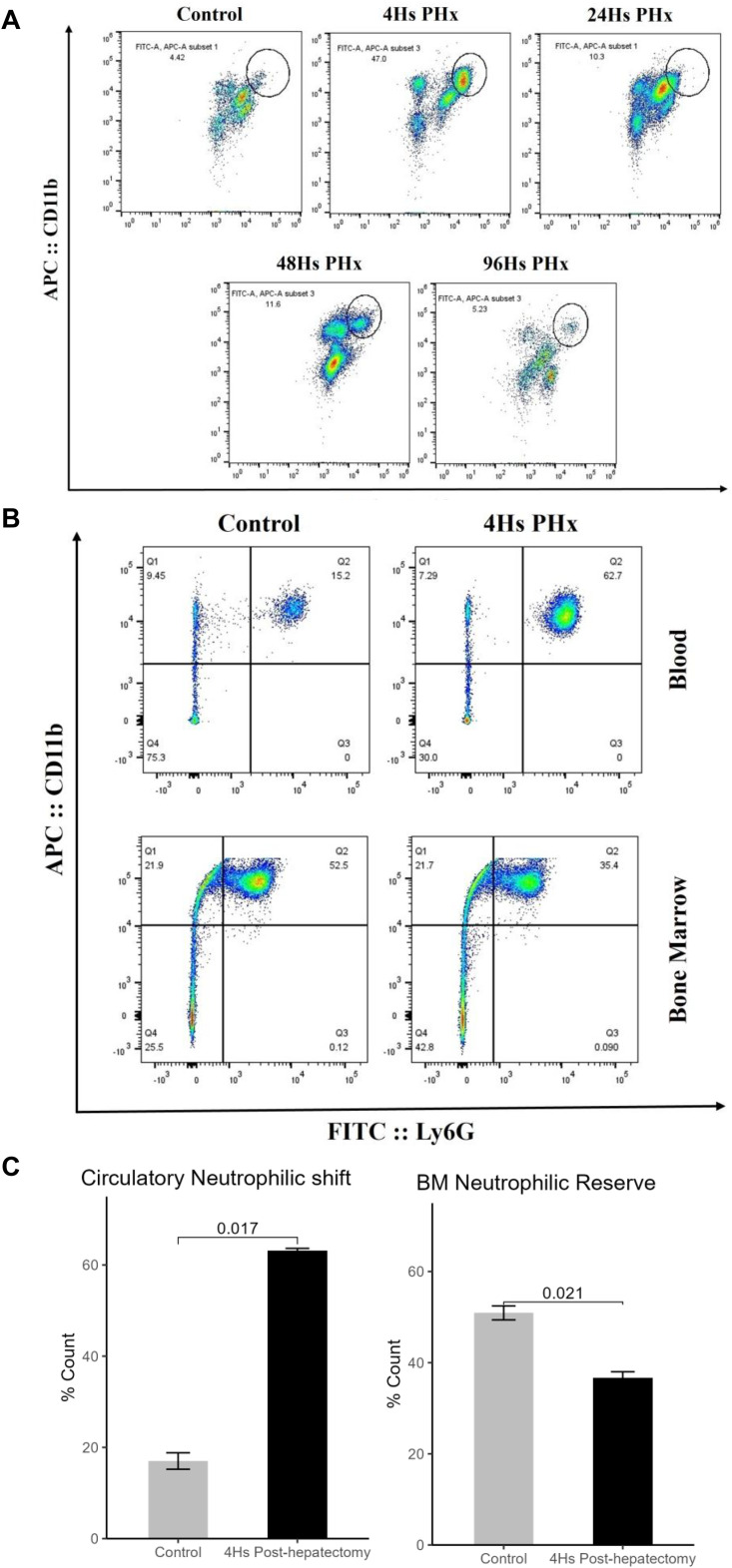
Quantitative analysis of neutrophil infiltration after hepatectomy II. (A) Flow cytometry showing quantitative changes in neutrophils at 4, 24, 48, and 96 h after hepatectomy. (B) Flowcytometry dot plots and (C) bar plots of neutrophil density in the peripheral blood and bone marrow 4 h after hepatectomy (Student’s *t*-test).

Given the limited quantitative precision of IHC, flow cytometry was subsequently performed on immune cells isolated from the liver tissue PHx. Flow cytometric analysis confirmed a significant increase in neutrophil density at 4 h PHx (*p* < 0.001), while no neutrophilia was detected at 24, 48, or 96 h (Fig 4A). Consistent with these findings, concurrent blood and bone marrow analyses demonstrated transient neutrophilia in circulation and depletion of bone marrow neutrophil stores at the same early time point (Fig 4B–4C).

**Fig 5:**
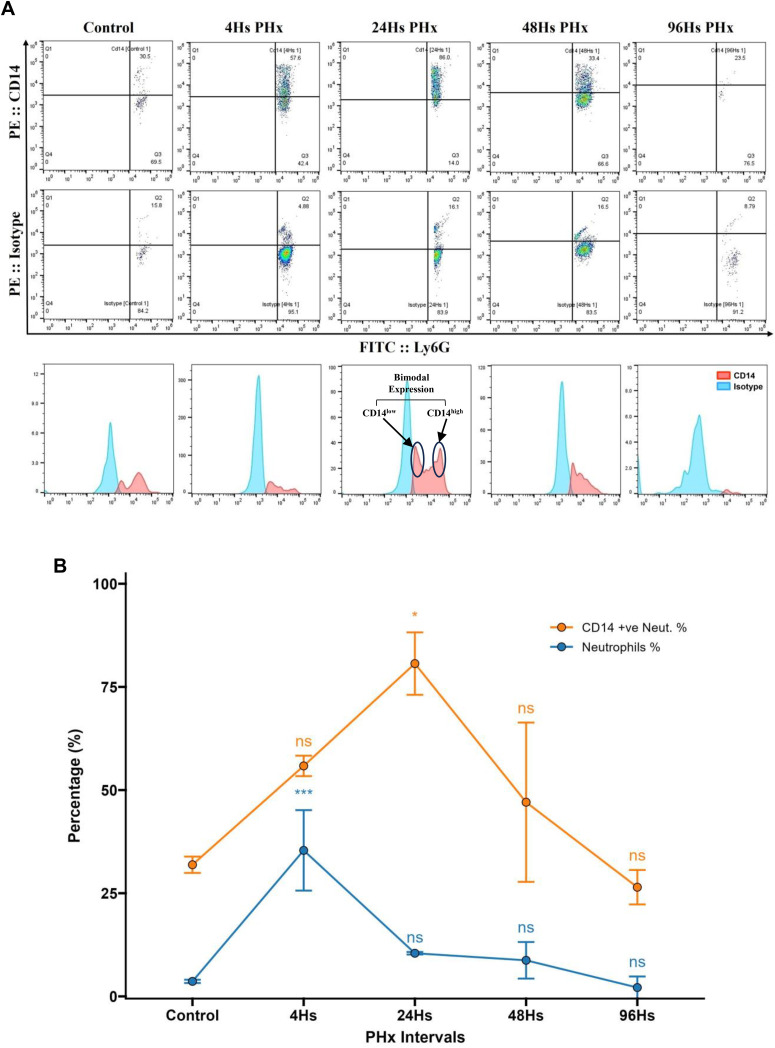
Phenotypic analysis of neutrophil CD14 expression after hepatectomy. (A) Flow cytometry of neutrophil CD14 expression at 4, 24, 48, and 96 h after hepatectomy (top panel, CD14; middle panel, isotype; lower panel, histogram showing both). (B) Diagram summarizing temporal variations in neutrophil density versus CD14 expression. Level of significance: * *p* = 0.012; *** *p* < 0.001 (one-way analysis of variance).

Further assessment of CD14^+^ neutrophils, validated by both isotype and fluorescence-minus-one controls, revealed a marked upregulation of CD14 protein expression at 24 h PHx (*p* = 0.012), whereas the 4-h samples showed only marginal changes, contrasting with the observed quantitative variations. Notably, histograms of CD14 fluorescence intensity exhibited a bimodal distribution across time points, indicating the presence of heterogeneous neutrophil subpopulations characterized by (CD14^high^) and (CD14^low^) expression levels. (Fig 5A). Collectively, these results suggest that neutrophil phenotypic modulation—reflected by CD14 expression—occurs independently of their quantitative changes during the regenerative response (Fig 5B).

## Discussion

The involvement of immune cells in the complex process of LR is well-recognized; however, the underlying mechanisms remain incompletely understood. In the present study, we observed marked hepatic neutrophil infiltration as early as 2–4 h PHx. This finding aligns with previous reports demonstrating rapid neutrophil recruitment within 2–3 h following sterile injury induced by laser irradiation or I/R, with infiltration typically resolving within 24 h [18]. The concurrent circulatory neutrophilia and depletion of bone marrow neutrophil stores observed here are consistent with the established paradigm of innate immune mobilization in response to acute stress in mice [19,20].CD14 is a well-established monocyte marker and a coreceptor for TLRs involved in mediating innate immune responses. Its expression, however, has also been reported in various somatic cells and other immune cells, including neutrophils [21]. In our study, scRNA-seq analysis identified *Cd14* among the top 10 markers of activated hepatic neutrophils. Consistent with this, most monocytes infiltrating the liver at 24 h PHx exhibited CD14^high^ phenotype, compared with only 6% of sessile resident hepatic macrophages [22]. Moreover, our integrated transcriptomic and protein-level analyses demonstrated phenotypic heterogeneity among neutrophils, characterized by CD14 upregulation at 24 h PHx coinciding with the resolution of neutrophilia and distinct from the early quantitative surge observed at 2–4 h. Notably, previous studies have reported that the 24-h time point marks the onset of the proliferative phase of regeneration, during which neutrophils adopt a pro-regenerative phenotype that promotes angiogenesis and hepatocyte proliferation following the initial inflammatory phase [8,9]. Enrichment of TLR4 and IL-6/JAK/STAT3 signaling pathways revealed by GSEA of *Ly6g–Cd14* gene pair, together with temporal coincidence, supports our hypothesis that CD14^high^ neutrophils constitute a pro-regenerative subset emerging during the proliferative phase PHx.

Numerous studies have highlighted the involvement of CD14 in tissue regeneration across various organs. For instance, an early study demonstrated that CD14^high^ monocytes induced by liver resection exhibit angiogenic potential and promote LR [23]. Umbilical cord-derived CD14^+^ monocytes have also been shown to exert neuroprotective effect by rescuing brain cells after vascular injury and are currently under clinical trials as a potential therapy for hypoxic–ischemic brain injury [24]. Beyond immune cells, peripheral blood CD14^+^ endothelial progenitor cells display stem cell-like properties and have emerged as a promising therapeutic option for ischemic conditions such as myocardial infarction [25]. Similarly, keratinocytic CD14 has been shown to be essential for double-stranded noncoding RNA-induced skin regeneration via TLR3 signaling, with *Cd14*-knockout mice exhibiting delayed wound healing compared with WT controls [26]. However, previous work reported that single knockouts of *Cd14*, *Tlr2*, or *Tlr4* produced only modest or inconsistent effects on LR [27], suggesting potential compensatory mechanisms. We speculate that the pro-regenerative effects of CD14 may be functionally compensated by other pathways, particularly when TLR signaling remains intact.

Retinoic acid, the active metabolite of vitamin A, has been reported to induce *Cd14* expression in keratinocytes at both transcript and protein levels [26]. Similarly, all-trans retinoic acid upregulates CD14 in human monocyte THP-1 cells [28]. Notably, retinoic acid also accelerates LR by promoting hepatocyte cell cycle progression [29]. Taken together, our findings suggest that the CD14^high^ neutrophil phenotype represents a pro-regenerative subset emerging PHx, potentially acting through enrichment of TLR4, and IL-6/JAK/STAT3 signaling pathways. Thus, we speculate that modulation of this via retinoic acid may represent a promising therapeutic strategy to enhance LR.

## Supporting information

S1 Fig Bioinformatics supplementary data I. (A) Dot plot of cell markers identifying each cluster. (B) Fluorescence-activated cell sorting gating strategy to analyze neutrophil phenotypes. [FMO: fluorescence minus one].
(TIF)
